# Exploring lifestyle activities as possible protective factors for life satisfaction: a cross-sectional study

**DOI:** 10.1186/s12877-025-06475-7

**Published:** 2025-10-27

**Authors:** Fumio Sakimoto, Takehiko Doi, Osamu Katayama, Soichiro Matsuda, Keitaro Makino, Hiroyuki Shimada

**Affiliations:** 1 Department of Preventive Gerontology, Center for Gerontology and Social Science, Research Institute, National Center for Geriatrics and Gerontology, 7-430, Morioka-Cho, Obu, Aichi 474-8511 Japan; 2 Center for Environmental and HealthSciences, Hokkaido University, North-12, West-7, Kita-ku, Sapporo, Hokkaido 060-0812 Japan

**Keywords:** Life satisfaction, Physical activity, Cognitive activity, Social activity

## Abstract

**Background and objective:**

Life satisfaction is an important factor in maintaining or increasing healthy life expectancy. However, as it is unclear what activities are associated with life satisfaction in older adults, we investigated whether lifestyle activity engagement contributes to higher life satisfaction levels.

**Methods:**

The study participants were 4,167 community-dwelling older adults aged 60 years or above in a cross-sectional survey. Life satisfaction was assessed using the Life Satisfaction Scale, which contains 13 questions, each comprising four levels. A 36-item Lifestyle Activities Questionnaire was used to assess participants’ lifestyle activities, with 12 items each covering physical, cognitive, and social activities. The results of each activity were categorized into high-, moderate-, or low-activity tertiles to determine their association with life satisfaction. We also investigated the relationship between life satisfaction and each activity type, stratifying the analysis by associated characteristics (i.e., living alone, gender, and age) using logistic regression, both before and after data imputation.

**Results:**

After imputation, the logistic regression analysis adjusted for covariates showed that physical, cognitive, and social activities had protective effects on life satisfaction. The high-activity group had a lower odds ratio (OR) for low life satisfaction compared with the low-activity group (physical activity: moderate group OR 0.91, 95% confidence intervals (CI) 0.77–1.08, high group OR 0.80, 95% CI 0.68–0.95; cognitive activity: moderate group OR 1.00, 95% CI 0.85–1.18, high group OR 0.71, 95% CI 0.59–0.85; social activity: moderate group OR 0.69, 95% CI 0.59–0.82, high group OR 0.66, 95% CI 0.55–0.78). The stratified analysis revealed different associations under each stratum, with only social activities demonstrating a protective effect on life satisfaction across all strata.

**Conclusion:**

High engagement in lifestyle activities had a protective effect on life satisfaction. The differences in the relationship between each activity type and life satisfaction varied based on participants’ characteristics. To maintain life satisfaction, it is necessary to understand the contribution of different activity types and the individual characteristics of the target population.

**Supplementary Information:**

The online version contains supplementary material available at 10.1186/s12877-025-06475-7.

## Background

Life satisfaction is closely related to increased healthy life expectancy among older adults [1, 2]. Life satisfaction is characterized by the presence of positive emotions and moods, the absence of negative emotions, a sense of fulfillment, and positive functioning [3]. Several factors have been reported to be associated with life satisfaction, including age, gender, cultural background, and social networks [2, 4–6]. Additionally, a decline in life satisfaction has been associated with activity limitations, depressive symptoms, and physical disabilities in older adults [7]. Moreover, low life satisfaction is also known to be associated with and predictive of disability [8, 9]. Therefore, preventing a decline in life satisfaction among older adults is an important public health issue.

While physical activity is associated with life satisfaction, the strength of this association may depend on activity intensity [10]. A systematic review suggested that even 10 min of physical activity per week can increase happiness [11]. Various cultural activities are also associated with life satisfaction. This is particularly true for women who participate in meetings, music, singing, theater, outdoor activities, dancing, and exercise or sports, while the same applies for men who actively participate in outdoor activities, dancing, and exercise or sports [12]. Engagement in social and leisure activities is also notably associated with increased life satisfaction [13, 14].

While the literature has explored the link between various activities and life satisfaction, the specific ways in which different activities influence this relationship, as well as the moderating role of individual characteristics, remain understudied. This study seeks to address this gap by investigating the relationship between participation in diverse life activities and life satisfaction among community-dwelling older adults, stratifying participants based on their individual characteristics.

## Materials and methods

### Participants

This study was conducted as a cross-sectional study. The target population included 4,167 community-dwelling older adults living in Takahama City, Aichi Prefecture, between September 2015 and February 2017 and aged 60 years or older at the time of the survey. The relevant data were obtained from the National Center for Geriatrics Study and Gerontology Syndrome (NCGG-SGS) [15], a cohort study that establishes a screening system for geriatric syndromes and validates evidence-based interventions focused on preventing these syndromes. The study included face-to-face interviews and assessments of physical and cognitive function. To accurately evaluate the outcomes of this study, the exclusion criteria were: (1) included individuals with a history of dementia (*n* = 7), depression (*n* = 109), or Parkinson's disease (*n* = 17) [16, 17] and (2) providing responses with missing data related to life satisfaction (*n* = 23), physical activity (*n* = 30), cognitive activity (*n* = 26), social activity (*n* = 24), living alone or not living alone (*n* = 1), years of education (*n* = 1), number of medications (*n* = 3), heart disease (*n* = 2), hypertension (*n* = 1), hyperlipidemia (*n* = 1), Mini-Mental State Examination (MMSE) (*n* = 20), and 15–item Geriatric Depression Scale (GDS-15) (*n* = 5).

Multiple imputation was applied to the relevant variables of 270 participants with missing data. Fifty imputed datasets were created to account for the uncertainty introduced by the missing values [18]. The final analysis included 3,897 participants before and 4,032 participants after multiple imputations. This study was approved by the Ethics Committee of the National Center for Geriatrics and Gerontology (approval number: 1440–5).

### Measurements

#### Measurement of life satisfaction

Life satisfaction was assessed using the Life Satisfaction Scale (LSS), which has five components: health, community, relationships, society, and social roles [19]. The LSS contains 13 items, with each question scored on a four-point Likert scale. The response options were poor = 1, not very good = 2, good = 3, and excellent = 4. The LSS was calculated by summing all responses (13–52 points), with higher scores indicating higher life satisfaction. The following questions were used to assess participants’ health status: (1) “How satisfied are you with your mental health?” and (2) “How satisfied are you with your physical health?”. The suitability of participants’ community environment and personal circumstances was measured by the questions: (3) “How satisfied are you with your housing?”; (4) “How satisfied are you with your community environment or neighborhood?”; and (5) “How satisfied are you with the condition of your household finances?”. Interpersonal relationship satisfaction was measured by the questions: (6) “How satisfied are you with your relationships with your family members?”; (7) “How satisfied are you with your relationships with your friends?”; and (8) “How satisfied are you with your relationships with your neighbors?”. Participants’ satisfaction with Japanese society as a whole was measured using the following questions: (9) “How satisfied are you with Japanese social security services, such as pension and insurance schemes?” and (10) “How satisfied are you with Japanese politics?”. Satisfaction with social roles was assessed and measured using the following questions: (11) “How satisfied are you with your own social role?”; (12) “How satisfied are you with your own accomplishments?”; and (13) “How satisfied are you with the amount of free time you have for yourself outside of work or carrying out household chores?”.

#### Measurement of lifestyle activity

The Lifestyle Activities Questionnaire (LAQ) was used to evaluate participants’ lifestyle activities. This questionnaire consists of 36 items covering physical, cognitive, and social activities [20]. Items relating to physical activity included walking, cycling, jogging, swimming, strength training, yoga, gymnastics, dancing, hiking, playing golf, and participating in ball sports. Cognitive activities included writing letters or keeping a diary, reading books, and reading magazines or newspapers. Learning activities included using a computer (including for Internet use), doing crossword puzzles, playing board games (e.g., card games, Go, or Japanese chess), playing musical instruments, participating in handicrafts, listening to music, appreciating art, and engaging with stocks or other forms of financial investment. Social activities included being officers of a senior club or neighborhood association, attending a regional event, engaging in environmental beautification activities, teaching-related activities, supporting older adults and children within and beyond the family, working (for pay), playing karaoke, eating with non-cohabiting family members, shopping with non-cohabiting family members, talking with friends and neighbors (including phone calls), attending events, and traveling. These 36 items focus on identifying the frequency of each activity over the past 12 months as follows: not at all = 0 points, less than once a month = 1 point, several times a month = 2 points, 1–2 times a week = 3 points, 3–6 times a week = 4 points, and daily = 5 points. The LAQ results were calculated by summing all responses (range: 0–60 points), with higher scores indicating higher levels of physical, cognitive, and social activity. The total scores for each activity were then divided into tertiles and operationally defined as the high-, moderate-, and low-activity groups [21].

#### Covariates

This study considered several potential covariates to examine the association between life satisfaction and lifestyle activities. The demographic characteristics examined comprised living alone, gender, age, years of education, chronic diseases, and medications, including those relating to heart disease, high blood pressure, diabetes, and hyperlipidemia, as well as cognitive impairment. MMSE scores were used for cognitive function [22], whereas GDS-15 scores were used to determine depressive symptoms [23].

### Statistical analyses

To verify the association between lifestyle activities and life satisfaction, the following procedure was followed. The Kolmogorov–Smirnov test was used to confirm normality. The LSS results were then classified into high- and low-satisfaction groups according to the median score of 39 points. The LSS score of 39 points has also been used in previous studies showing the cut-off point for depressive symptoms and the relationship between productive activities and life satisfaction [19, 24]. In the univariate analysis, the participants were classified according to whether they exhibited high or low levels of life satisfaction. Pearson’s χ^2^ test was used to analyze the categorical variables used in this study (gender, chronic diseases). Student’s t-test was used for continuous variables (age, year of education, and the number of medications) and the Mann–Whitney U test for ordinal variables (physical activity score, cognitive activity score, social activity score, MMSE, and GDS-15).

To use the multiple imputation (MI) method for missing data values, Little's Missing Completely At Random (MCAR) test was first performed to check the mechanism of missing data [25]. Once it was confirmed that the pattern of missing data was random, the following analyses were performed on the dataset imputed using MI and the dataset before imputation. The dependent variable was life satisfaction and the independent variables were all lifestyle activities for the high-, moderate-, and low-activity groups. Logistic regression analysis adjusted for covariates confirmed the protective role of each activity regarding life satisfaction. Stratified analyses were used to facilitate a better understanding of the relationship between life satisfaction and physical, cognitive, and social activities. Life satisfaction levels are strongly associated with living alone [4], gender [26], and age [5]. Therefore, the stratified analyses considered participants’ living status (whether living alone), gender (men or women), and age (75 > or ≤ 75). The estimated odds ratios (ORs) of protection against low life satisfaction were judged using 95% confidence intervals (CI). The significance level was set at *p* < 0.05. All analyses were performed using IBM Statistical Package for Social Sciences version 29.0 (IBM Japan, Tokyo, Japan).

## Results

Table 1 shows participants’ characteristics according to their reported life satisfaction before imputation. The mean age of the 3,897 participants (women: 2,157) was 71.3 ± 7.0 years and the mean years of education were 11.3 ± 2.4 years. Participants were classified as having high life satisfaction (2,007, 51.5%) or low life satisfaction (1,890, 48.5%). The high life satisfaction group included more women (58.1%), a higher lifestyle activity score (across the physical, cognitive, and social dimensions), a lower proportion of patients with diabetes, and a lower GDS-15 score than the low life satisfaction group.

**Table 1:** Participants’ characteristics based on life satisfaction classifications

	All participants	High life satisfaction	Low life satisfaction	*p*-value
	*n* = 3,897	*n* = 2,007	*n* = 1,890	
Physical activity, score	5.0 (2.0–9.0)	5.0 (3.0–10.0)	5.0 (2.0–8.0)	<.001
Cognitive activity, score	12.0 (8.0–17.0)	13.0 (8.0–18.0)	11.0 (7.0–15.0)	<.001
Social activity, score	8.0 (5.0–12.0)	9.0 (6.0–13.0)	7.0 (4.0–10.0)	<.001
Living alone	438 (11.2)	209 (10.4)	229 (12.1)	0.093
Sex (female)	2,157 (55.4)	1,166 (58.1)	991 (52.4)	<.001
Age in years	71.3 ± 7.0	72.1 ± 7.0	70.6 ± 6.9	0.464
Years of education	11.3 ± 2.7	11.3 ± 2.4	11.2 ± 2.4	0.369
Number of medications	2.0 (1.0–4.0)	2.7 ± 2.5	2.9 ± 2.8	0.068
Heart disease	599 (15.4)	320 (15.9)	279 (14.8)	0.306
Hypertension	1,853 (47.5)	982 (48.9)	871 (46.1)	0.076
Diabetes	559 (14.3)	242 (12.1)	317 (16.8)	<.001
Hyperlipemia	1,131 (29.0)	591 (29.4)	540 (28.6)	0.547
MMSE	28.0 (25.0–29.0)	28.0 (25.0–29.0)	28.0 (25.0–29.0)	0.485
GDS-15	2.0 (1.0–4.0)	2.0 (10.−3.0)	3.0 (2.0–6.0)	<.001

Values are expressed as mean ± SD; median (interquartile range); or N (%)

**Fig. 1:**
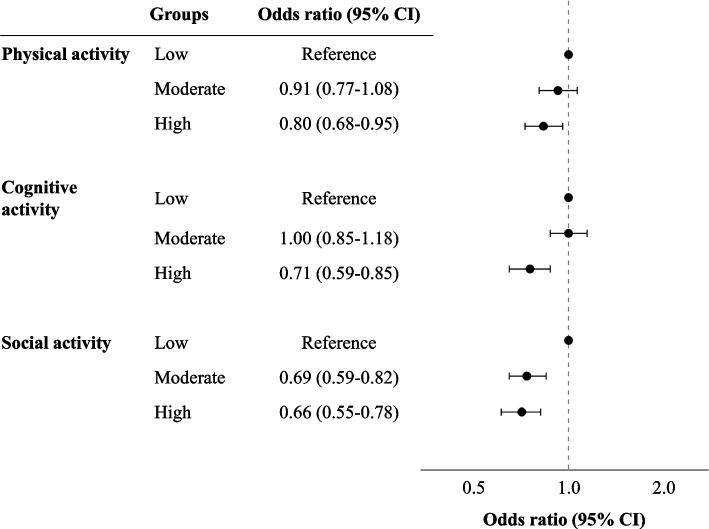
The association between life satisfaction and lifestyle activities after imputation. The OR of each lifestyle activity group was adjusted for covariates, with moderate-, high- and low-activity groups subsequently identified. Participants were classified into one of these groups according to their tertile scores (low, moderate, and high). High activity was shown to be more protective of life satisfaction than low activity engagement across the physical, cognitive, and social activity categories

The LAQ scores were categorized into three groups for each activity based on the following tertiles: for physical activity—low group 0–3, moderate group 4–7, high group ≥ 7; for cognitive activity—low group 0–10, moderate group 11–17, high group ≥ 18; and for social activity—low group 0–6, moderate group 7–10, high group ≥ 11. Logistic regression analysis showed that, compared to the low-activity group for physical activity, cognitive activity, and social activity, the high-activity group had a protective association with life satisfaction both before and after the imputation, when adjusted for covariates (Fig. 1, Supplementary Tables 1 and 2).

**Fig. 2:**
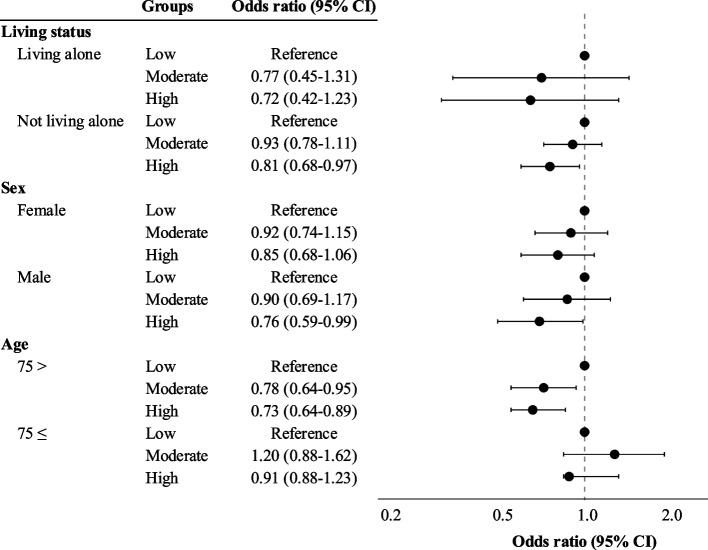
The association between life satisfaction and physical activity after stratification and imputation. High levels of physical activity were associated with a protective effect on life satisfaction among older adults who did not live alone, as well as those living alone, males, and participants aged 74 and under

**Fig. 3:**
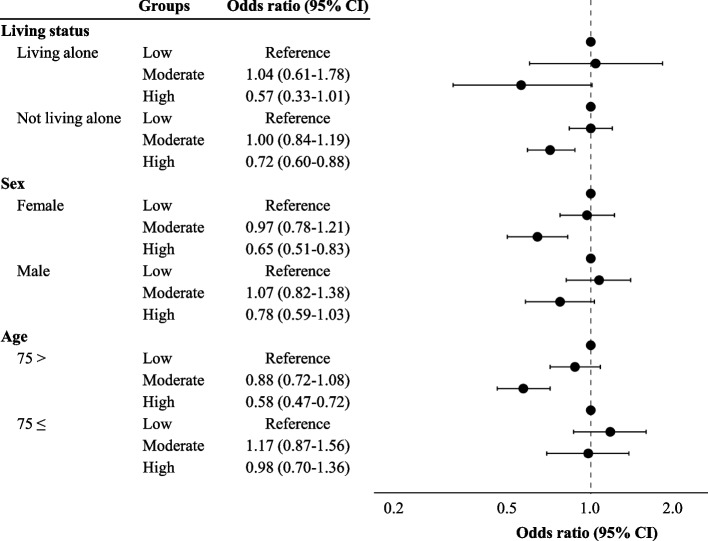
The association between life satisfaction and cognitive activity after stratification and imputation. High-level cognitive activity was associated with a protective effect on life satisfaction among older adults who were not living alone, as well as among women and participants aged 74 and under

**Fig. 4:**
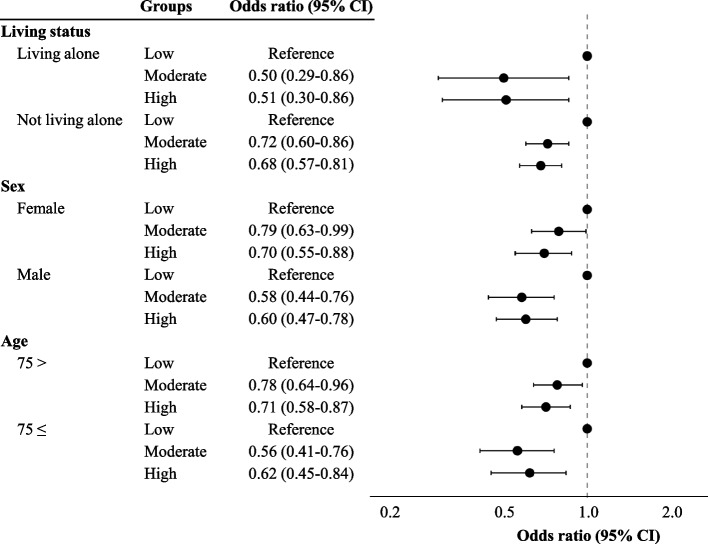
The association between life satisfaction and social activity after stratification and imputation. In all the stratified analyses, high levels of social activity were found to be associated with a protective effect on life satisfaction

After imputation, the relationship between life satisfaction and physical, cognitive, and social activities in the stratified analyses is shown in Figs. 2, 3, and 4. In the stratified analysis, not living alone, being male, and being 74 years old or younger were associated with physical activity and life satisfaction (Fig. 2 and Supplementary Table 4). Before imputation, being male was not associated with physical activity and life satisfaction (Supplementary Table 3). In terms of cognitive activities, not living alone, being female, and being 74 years old or younger were all associated with life satisfaction, before and after the imputation (Fig. 3, Supplementary Tables 5 and 6). Concerning social activities, a protective association was found between life satisfaction and all individual characteristics stratified, both before and after the imputation (Fig. 4, Supplementary Tables 7 and 8).

## Discussion

Our study revealed that the strength of the relationship between activities and life satisfaction varies by activity type. For lifestyle activities, being a member of the high-activity group had a protective association with life satisfaction. In the stratified analysis of the data set after imputation, those who engaged in higher levels of physical activity had a protective association with life satisfaction in terms of not living alone, being male, and being 74 years old or younger. Engagement cognitive activity was a protective association between living alone, being female, and being under 74 years old and life satisfaction. The association between social activity and life satisfaction was not dependent on the individual participant characteristics.

Social activities were associated with increased life satisfaction across all activity groups. This provides strong support for the importance of social interaction for older adults [2]. Physical and cognitive activities may also be related to higher life satisfaction. In a previous study, participants who engaged in sports/games reported higher levels of life satisfaction than those who did not; however, no such association was found regarding participation in music, art, drama, or reading [27]. This report was based on whether participants had engaged in any of these activities during the past week and did not consider the frequency of their participation. The extent to which individuals engage in various activities thus seems to affect their life satisfaction.

A systematic review reported that physical activity has a positive effect on life satisfaction [11], suggesting that any form of physical activity may have a positive impact on healthy aging [28]. Moreover, engaging in regular exercise or physical activity is significantly associated with increased life satisfaction among older adults [29]. However, the intensity of the physical activity may also affect life satisfaction. A previous study among older adults reported that high-intensity physical activity (8 metabolic equivalents [METs]) was more strongly associated with higher satisfaction compared with low-intensity physical activity (3.3 METs) [30]. Furthermore, several reports disagree on gender differences [31, 32]. This implies that more research is necessary to elucidate the exact relationship between sex, the intensity, type, and frequency of physical activity, and life satisfaction.

Active leisure activities, such as participation in clubs and volunteering, were more strongly associated with life satisfaction compared with passive leisure activities [33]. Cognitive activities, including passive cognitive activities such as reading, were associated with increased life satisfaction, but not for participants aged 75 years and over. In general, well-being (life satisfaction) is U-shaped over the lifecycle, declining the most in middle age and increasing in older age [34]. However, the analyses using fixed effects models also suggested that the U-shaped transition disappears and life satisfaction tends to decline from the age of 75 years onward [35]. This finding provides further evidence that age must be considered when interpreting the effects of cognitive activities. Furthermore, it has been reported that gender differences affect participation in cognitive activities and are also related to health and satisfaction [12, 36]. It has also been pointed out that the higher the sense of loneliness, the lower the participation in cognitive activities [37]. The findings of the present study support these previous results.

Our findings indicate a positive association between higher levels of social activity and greater life satisfaction. This result is consistent with previous studies [1, 14, 27]. Notably, stratified analyses revealed consistent findings across different demographic groups. Given the well-established importance of social participation for the well-being of older adults living alone [4, 38], and the higher levels of life satisfaction reported by participants living with others [39, 40], our results suggest that social activity is a significant factor in life satisfaction, regardless of living arrangements [39, 40]. While previous research has suggested that women tend to have higher levels of life satisfaction [41], our stratified analysis by gender showed a positive association between social activity and life satisfaction in both men and women. Moreover, this positive association was observed across all levels of social activity, from moderate to high, among both men and women. High social activity was also associated with increased life satisfaction regardless of age, a finding that is corroborated by the results of several extant studies [2, 13]. These findings substantiate the notion that social engagement is a significant determinant of life satisfaction across the lifespan.

This study has a number of limitations and strengths as follows. First, in cross-sectional studies temporal concepts are difficult to describe, meaning the causality between lifestyle activities and life satisfaction cannot be clarified. Second, multiple life satisfaction ratings must be explored to ensure consistency with other ratings. Third, although stratified analyses were carried out, the interaction between variables was not examined. Notably, this study revealed an association between life satisfaction and lifestyle activities across a range of stratified analyses. The findings of these stratified analyses could be used in future studies to provide clearer conclusions with regard to the causality of our results.

## Conclusions

The results of this study suggest that greater engagement in physical, cognitive, and social activities is associated with greater life satisfaction. The stratified analyses provide new insights into the factors that determine life satisfaction, including the differences in the types of recommended activities and the characteristics of older adults. Future studies must consider approaches that consider different lifestyle activities and individual characteristics, which may help extend healthy life expectancy among older adults. Further research is thus needed to capture long-term changes in lifestyle activities and the corresponding impact on life satisfaction.

## Supplementary Information

Supplementary Material 1: Supplementary Table 1. Odds of life satisfaction based on physical, cognitive, and social activities before imputation. Supplementary Table 2. Odds of life satisfaction based on physical, cognitive, and social activities after imputation. Supplementary Table 3. Odds of life satisfaction based on physical activity and stratified analysis before imputation. Supplementary Table 4. Odds of life satisfaction based on physical activity and stratified analysis after imputation. Supplementary Table 5. Odds of life satisfaction based on cognitive activity and stratified analysis before imputation. Supplementary Table 6. Odds of life satisfaction based on cognitive activity and stratified analysis after imputation. Supplementary Table 7. Odds of life satisfaction based on social activity and stratified analysis before imputation. Supplementary Table 8. Odds of life satisfaction based on social activity and stratified analysis after imputation.

## Data Availability

The datasets generated and/or analyzed during the current study are not publicly available due to not acquiring consent from participants to share datasets but are available from the corresponding author on reasonable request.

## Abbreviations

NCGG-SGS: National Center for Geriatrics Study and Gerontology Syndrome
MMSE: Mini-Mental State Examination
GDS-15: Geriatric Depression Scale
LSS: Life Satisfaction Scale
LAQ: The Lifestyle Activities Questionnaire
