# Supplementary material for: Exploring lifestyle activities as possible protective factors for life satisfaction: a cross-sectional study
Source: BMC Geriatr. 2025 Oct 27;25:812. doi: 10.1186/s12877-025-06475-7 (PMC12557914; doi:10.1186/s12877-025-06475-7)
Supplement: Supplementary file 1 — Supplementary Material 1: Supplementary Table 1. Odds of life satisfaction based on physical, cognitive, and social activities before imputation. Supplementary Table 2. Odds of life satisfaction based on physical, cognitive, and social activities after imputation. Supplementary Table 3. Odds of life satisfaction based on physical activity and stratified analysis before imputation. Supplementary Table 4. Odds of life satisfaction based on physical activity and stratified analysis after imputation. Supplementary Table 5. Odds of life satisfaction based on cognitive activity and stratified analysis before imputation. Supplementary Table 6. Odds of life satisfaction based on cognitive activity and stratified analysis after imputation. Supplementary Table 7. Odds of life satisfaction based on social activity and stratified analysis before imputation. Supplementary Table 8. Odds of life satisfaction based on social activity and stratified analysis after imputation. [file 12877_2025_6475_MOESM1_ESM.pdf]

Supplementary Table 1. Odds of life satisfaction based on physical, cognitive, and social activities before imputation.

	Physical activity		Cognitive activity		Social activity	
	OR (95% CI)	p value	OR (95% CI)	p value	OR (95% CI)	p value
Low	Reference		Reference		Reference	
Moderate	0.93 (0.79-1.11)	0.42	1.02 (0.86-1.21)	0.82	0.69 (0.58-0.82)	<.001
High	0.82 (0.69-0.97)	0.02	0.71 (0.59-0.85)	<.001	0.66 (0.55-0.78)	<.001
Not living alone	1.08 (0.86-1.36)	0.50	1.09 (0.87-1.38)	0.45	1.08 (0.86-1.36)	0.49
Gender (Male)	1.34 (1.16-1.55)	<.001	1.33 (1.15-1.54)	<.001	1.33 (1.15-1.54)	<.001
Age	0.94 (0.93-0.96)	<.001	0.94 (0.93-0.96)	<.001	0.94 (0.93-0.95)	<.001
Year of education	0.97 (0.94-1.00)	0.05	0.98 (0.95-1.01)	0.26	0.97 (0.94-1.01)	0.10
Number of medication	1.05 (1.01-1.09)	0.01	1.05 (1.02-1.09)	0.00	1.04 (1.01-1.08)	0.01
Heart disease	0.83 (0.68-1.02)	0.08	0.85 (0.69-1.04)	0.11	0.83 (0.68-1.02)	0.08
Hypertension	0.88 (0.75-1.02)	0.10	0.88 (0.75-1.03)	0.10	0.90 (0.77-1.04)	0.16
Diabetes	1.27 (1.03-1.57)	0.03	1.26 (1.02-1.55)	0.04	1.28 (1.03-1.58)	0.03
Hyperlipidemia	0.92 (0.78-1.08)	0.30	0.91 (0.78-1.08)	0.28	0.92 (0.78-1.08)	0.29
MMSE	1.03 (1.00-1.06)	0.06	1.03 (1.00-1.03)	0.03	1.03 (1.00-1.06)	0.07
GDS-15	1.44 (1.39-1.49)	<.001	1.44 (1.39-1.49)	<.001	1.42 (1.38-1.47)	<.001

Supplementary Table 2. Odds of life satisfaction based on physical, cognitive, and social activities after imputation.

	Physical activity		Cognitive activity		Social activity	
	OR (95% CI)	p value	OR (95% CI)	p value	OR (95% CI)	p value
Low	Reference		Reference		Reference	
Moderate	0.91 (0.77-1.08)	0.28	1.00 (0.85-1.18)	0.99	0.69 (0.59-0.82)	<.001
High	0.80 (0.68-0.95)	0.01	0.71 (0.59-0.85)	<.001	0.66 (0.55-0.78)	<.001
Not living alone	1.06 (0.85-1.33)	0.61	1.07 (0.85-1.34)	0.57	1.06 (0.85-1.33)	0.61
Gender (Male)	1.33 (1.16-1.54)	<.001	1.32 (1.15-1.53)	<.001	1.32 (1.14-1.52)	<.001
Age	0.94 (0.93-0.96)	<.001	0.94 (0.93-0.96)	<.001	0.94 (0.93-0.95)	<.001
Year of education	0.97 (0.94-1.00)	0.04	0.98 (0.95-1.01)	0.23	0.97 (0.94-1.00)	0.09
Number of medication	1.05 (1.01-1.08)	0.01	1.05 (1.01-1.08)	0.01	1.04 (1.01-1.08)	0.02
Heart disease	0.87 (0.72-1.07)	0.19	0.89 (0.73-1.08)	0.24	0.88 (0.72-1.07)	0.20
Hypertension	0.90 (0.78-1.05)	0.18	0.90 (0.78-1.05)	0.18	0.92 (0.79-1.07)	0.26
Diabetes	1.24 (1.00-1.52)	0.05	1.22 (0.99-1.51)	0.06	1.24 (1.01-1.53)	0.04
Hyperlipidemia	0.93 (0.79-1.08)	0.34	0.92 (0.79-1.08)	0.32	0.92 (0.79-1.08)	0.33
MMSE	1.03 (1.00-1.06)	0.07	1.03 (1.00-1.07)	0.03	1.03 (1.00-1.06)	0.08
GDS-15	1.43 (1.38-1.48)	<.001	1.43 (1.39-1.48)	<.001	1.41 (1.37-1.46)	<.001

Supplementary Table 3. Odds of life satisfaction based on physical activity and stratified analysis before imputation.

	Living status				Sex				Age			
	Living alone		Not living alone		Female		Male		Age 75>		Age 75≦	
	OR (95% CI)	p value	OR (95% CI)	p value	OR (95% CI)	p value	OR (96% CI)	p value	OR (95% CI)	p value	OR (96% CI)	p value
**Physical activity**												
Low	Reference		Reference		Reference		Reference		Reference		Reference	
Moderate	0.78 (0.46–1.35)	0.38	0.95 (0.79–1.14)	0.57	0.93 (0.74–1.16)	0.52	0.94 (0.72–1.23)	0.67	0.79 (0.65–0.97)	0.02	1.26 (0.93–1.72)	0.14
High	0.69 (0.40–1.19)	0.18	0.83 (0.69–0.99)	0.04	0.85 (0.68–1.07)	0.17	0.79 (0.72–1.03)	0.08	0.74 (0.60–0.90)	0.00	0.95 (0.70–1.28)	0.73
Not living alone					1.05 (0.79–1.38)	0.74	1.19 (1.72–1.80)	0.41	1.01 (0.75–1.36)	0.95	1.11 (0.78–1.59)	0.57
Gender (Male)	1.69 (1.02–2.81)	0.04	1.32 (1.13–1.54)	<.001					1.29 (1.08–1.53)	0.01	1.50 (1.15–1.95)	0.00
Age	0.96 (0.92–0.99)	0.01	0.94 (0.93–0.95)	<.001	0.94 (0.92–0.95)	<.001	0.95 (0.72–0.97)	<.001				
Year of education	0.97 (0.87–1.07)	0.50	0.97 (0.94–1.00)	0.06	0.99 (0.94–1.04)	0.63	0.95 (0.72–0.99)	0.02	0.99 (0.95–1.02)	0.46	1.00 (0.94–1.06)	0.87
Number of medication	1.07 (0.97–1.19)	0.17	1.05 (1.01–1.08)	0.01	1.05 (1.01–1.10)	0.03	1.05 (1.72–1.10)	0.10	1.03 (0.98–1.07)	0.227	1.04 (0.99–1.10)	0.102
Heart disease	0.80 (0.43.149)	0.48	0.84 (0.67–1.04)	0.11	0.88 (0.67–1.18)	0.40	0.78 (0.72–1.05)	0.10	0.76 (0.58–1.00)	0.05	0.88 (0.64–1.20)	0.40
Hypertension	0.66 (0.41–1.06)	0.09	0.91 (0.77–1.07)	0.24	0.89 (0.73–1.10)	0.28	0.89 (0.72–1.12)	0.33	0.81 (0.67–0.98)	0.03	0.97 (0.75–1.27)	0.85
Diabetes	1.14 (0.57–2.29)	0.71	1.28 (1.02–1.60)	0.03	1.01 (0.76–1.36)	0.94	1.63 (1.72–2.22)	0.00	1.40 (1.08–1.83)	0.01	1.11 (0.78–1.58)	0.56
Hyperlipidemia	0.98 (0.60.158)	0.92	0.91 (0.77–1.08)	0.29	1.03 (0.84–1.27)	0.74	0.76 (1.72–1.00)	0.05	0.88 (0.72–1.07)	0.19	1.07 (0.81–1.42)	0.63
MMSE	1.05 (0.951–115)	0.36	1.03 (1.00–1.06)	0.09	1.00 (0.96–1.04)	0.97	1.07 (1.72–1.12)	0.01	1.06 (1.02–1.11)	0.00	1.02 (0.97–1.06)	0.51
GDS–15	1.45 (1.32–1.60)	<.001	1.44 (1.39–1.49)	<.001	1.42 (1.35–1.48)	<.001	1.48 (1.72–1.56)	<.001	1.47 (1.40–1.53)	<.001	1.38 (1.31–1.45)	<.001

Supplementary Table 4. Odds of life satisfaction based on physical activity and stratified analysis after imputation.

	Living status				Sex				Age			
	Living alone		Not living alone		Female		Male		Age 75>		Age 75≦	
	OR (95% CI)	p value	OR (95% CI)	p value	OR (95% CI)	p value	OR (96% CI)	p value	OR (95% CI)	p value	OR (96% CI)	p value
**Physical activity**												
Low	Reference		Reference		Reference		Reference		Reference		Reference	
Moderate	0.77 (0.45–1.31)	0.33	0.93 (0.78–1.11)	0.42	0.92 (0.74–1.15)	0.47	0.90 (0.69–1.17)	0.44	0.78 (0.64–0.95)	0.01	1.20 (0.88–1.62)	0.25
High	0.72 (0.42–1.23)	0.23	0.81 (0.68–0.97)	0.02	0.85 (0.68–1.06)	0.15	0.76 (0.59–0.99)	0.04	0.73 (0.64–0.89)	0.00	0.91 (0.88–1.23)	0.55
Not living alone					1.04 (0.79–1.37)	0.78	1.13 (0.75–1.69)	0.56	0.99 (1.64–1.32)	0.93	1.10 (9.88–1.56)	0.61
Gender (Male)	1.57 (0.95–2.60)	0.08	1.32 (1.14–1.53)	<.001					1.28 (1.08–1.52)	0.01	1.50 (0.88–1.94)	0.00
Age	0.96 (0.92–0.99)	0.01	0.94 (0.93–0.95)	<.001	0.94 (0.92–0.95)	<.001	0.95 (0.94–0.97)	<.001				
Year of education	0.98 (0.89–1.08)	0.68	0.97 (0.93–1.00)	0.04	0.99 (0.94–1.04)	0.73	0.95 (0.91–0.99)	0.012	0.99 (0.64–1.02)	0.44	0.99 (4.88–1.05)	0.78
Number of medication	1.06 (0.96–1.17)	0.24	1.04 (1.01–1.08)	0.02	1.05 (1.00–1.10)	0.03	1.04 (0.99–1.09)	0.15	1.03 (1.64–1.07)	0.25	1.04 (2.88–1.09)	0.15
Heart disease	0.85 (0.46–1.57)	0.60	0.88 (0.71–1.08)	0.23	0.93 (0.71–1.23)	0.63	0.82 (0.61–1.10)	0.18	0.80 (0.64–1.04)	0.09	0.93 (0.88–1.26)	0.65
Hypertension	0.73 (0.46–1.17)	0.19	0.92 (0.79–1.08)	0.32	0.91 (0.74–1.12)	0.37	0.92 (0.73–1.16)	0.48	0.82 (0.64–0.99)	0.03	1.04 (0.88–1.35)	0.76
Diabetes	1.06 (0.54–2.09)	0.86	1.25 (1.01–1.56)	0.05	0.99 (0.74–1.31)	0.92	1.58 (1.16–2.15)	0.00	1.33 (0.64–1.73)	0.03	1.15 (0.88–1.63)	0.43
Hyperlipidemia	0.93 (0.58–1.50)	0.78	0.92 (0.78–1.09)	0.36	1.03 (0.84–1.26)	0.79	0.79 (0.60–1.02)	0.07	0.90 (1.64–1.09)	0.27	1.04 (2.88–1.37)	0.76
MMSE	1.04 (0.95–1.15)	0.42	1.03 (1.00–1.06)	0.09	1.00 (0.96–1.04)	0.96	1.06 (1.02–1.11)	0.01	1.06 (1.64–1.10)	0.01	1.02 (0.88–1.07)	0.35
GDS-15	1.46 (1.32–1.61)	<.001	1.43 (1.38–1.48)	<.001	1.41 (1.35–1.47)	<.001	1.46 (1.39–1.54)	<.001	1.46 (1.64–1.52)	<.001	1.37 (0.88–1.44)	<.001

Supplementary Table 5. Odds of life satisfaction based on cognitive activity and stratified analysis before imputation.

	Living status				Sex				Age			
	Living alone		Not living alone		Female		Male		Age 75>		Age 75≦	
	OR (95% CI)	p value	OR (95% CI)	p value	OR (95% CI)	p value	OR (96% CI)	p value	OR (95% CI)	p value	OR (96% CI)	p value
**Cognitive activity**												
Low	Reference		Reference		Reference		Reference		Reference		Reference	
Moderate	1.02 (0.86–1.22)	.806	1.04 (0.60–1.78)	0.90	1.01 (0.81–1.26)	0.92	1.05 (0.81–1.37)	0.70	0.88 (0.72–1.08)	0.24	1.24 (0.93–1.67)	0.15
High	0.73 (0.60–0.89)	.002	0.52 (0.29–0.93)	0.03	0.66 (0.52–0.85)	0.00	0.77 (0.58–0.01)	0.06	0.58 (0.47–0.73)	<.001	0.98 (0.70–1.37)	0.90
Not living alone					1.06 (0.80–1.40)	0.68	1.21 (0.80–1.84)	0.36	1.02 (0.76–1.38)	0.88	1.11 (0.78–1.58)	0.57
Gender (Male)	1.31 (1.12–1.52)	<.001	1.69 (1.01–2.81)	0.05					1.27 (1.06–1.51)	0.01	1.49 (1.15–1.94)	0.00
Age	0.94 (0.93–0.95)	<.001	0.96 (0.92–0.99)	0.01	0.94 (0.92–0.95)	<.001	0.95 (0.94–0.97)	<.001				
Year of education	0.98 (0.95–1.01)	.238	1.00 (0.90–1.12)	0.96	1.01 (0.96–1.06)	0.81	0.96 (0.92–1.00)	0.06	1.01 (0.97–1.05)	0.69	1.00 (0.94–1.06)	0.903
Number of medication	1.05 (1.01–1.08)	.013	1.09 (0.99–1.21)	0.09	1.05 (1.01–1.10)	0.03	1.05 (0.99–1.10)	0.09	1.03 (0.99–1.08)	0.19	1.05 (0.99–1.10)	0.08
Heart disease	0.85 (0.68–1.05)	.138	0.82 (0.44–1.53)	0.53	0.90 (0.68–1.20)	0.49	0.79 (0.59–0.07)	0.13	0.78 (0.59–1.02)	0.07	0.87 (0.64–1.19)	0.38
Hypertension	0.91 (0.77–1.07)	.254	0.62 (0.38–1.02)	0.06	0.90 (0.73–1.10)	0.30	0.89 (0.71–0.12)	0.32	0.82 (0.68–0.99)	0.04	0.97 (0.74–1.26)	0.82
Diabetes	1.27 (1.01–1.58)	.038	1.13 (0.57–2.24)	0.74	0.99 (0.74–1.33)	0.96	1.63 (1.19–0.23)	0.00	1.41 (1.08–1.84)	0.01	1.11 (0.78–1.58)	0.57
Hyperlipidemia	0.91 (0.76–1.08)	.271	0.96 (0.59–1.56)	0.86	1.03 (0.84–1.26)	0.80	0.76 (0.58–1.00)	0.05	0.86 (0.71–1.06)	0.15	1.07 (0.81–1.42)	0.63
MMSE	1.03 (1.00–1.07)	.054	1.07 (0.97–1.18)	0.20	1.00 (0.96–1.05)	0.88	1.07 (1.03–1.12)	0.00	1.07 (1.03–1.12)	<.001	1.02 (0.97–1.06)	0.52
GDS–15	1.44 (1.39–1.49)	<.001	1.44 (1.31–1.59)	<.001	1.41 (1.35–1.48)	<.001	1.48 (1.41–1.56)	<.001	1.47 (1.40–1.53)	<.001	1.38 (1.31–1.46)	<.001

Supplementary Table 6. Odds of life satisfaction based on cognitive activity and stratified analysis after imputation.

	Living status				Sex				Age			
	Living alone		Not living alone		Female		Male		Age 75>		Age 75≦	
	OR (95% CI)	p value	OR (95% CI)	p value	OR (95% CI)	p value	OR (96% CI)	p value	OR (95% CI)	p value	OR (96% CI)	p value
**Cognitive activity**												
Low	Reference		Reference		Reference		Reference		Reference		Reference	
Moderate	1.04 (0.61–1.78)	0.88	1.00 (0.84–1.19)	0.99	0.97 (0.78–1.21)	0.79	1.07（0.82–1.38）	0.63	0.88 (0.72–1.08)	0.23	1.17 (0.87–1.56)	0.30
High	0.57 (0.33–1.01)	0.05	0.72 (0.60–0.88)	<.001	0.65 (0.51–0.83)	<.001	0.78 （0.59–1.03）	0.08	0.58 (0.47–0.72)	<.001	0.98 (0.70–1.36)	0.88
Not living alone					1.05 (0.80–1.38)	0.72	1.14 （0.76–1.72）	0.52	1.00 (0.74–1.33)	0.98	1.09 (0.77–1.55)	0.62
Gender (Male)	1.56 (0.95–2.57)	0.08	1.31 (1.13–1.52)	<.001					1.27 (1.07–1.51)	0.01	1.48 (1.15–1.92)	0.00
Age	0.96 (0.92–0.99)	0.01	0.94 (0.93–0.95)	<.001	0.94 (0.92–0.95)	<.001	0.95 （0.94–0.97）	<.001				
Year of education	1.01 (0.91–1.13)	0.79	0.98 (0.94–1.01)	0.18	1.01 (0.96–1.06)	0.69	0.95 （0.91–1.00）	0.03	1.01 (0.97–1.05)	0.73	0.99 (0.94–1.05)	0.82
Number of medication	1.08 (0.97–1.19)	0.15	1.04 (1.01–1.08)	0.02	1.05 (1.01–1.10)	0.03	1.04 （0.99–1.09）	0.14	1.03 (0.98–1.07)	0.22	1.04 (0.99–1.09)	0.13
Heart disease	0.87 (0.47–1.61)	0.66	0.89 (0.72–1.10)	0.28	0.95 (0.72–1.26)	0.73	0.83 （0.62–1.11）	0.21	0.81 (0.62–1.06)	0.12	0.93 (0.68–1.26)	0.62
Hypertension	0.70 (0.43–1.13)	0.14	0.93 (0.79–1.09)	0.34	0.91 (0.75–1.12)	0.38	0.92 （0.73–1.16）	0.48	0.82 (0.68–0.99)	0.04	1.03 (0.80–1.34)	0.80
Diabetes	1.05 (0.54–2.05)	0.89	1.24 (1.00–1.55)	0.05	0.97 (0.72–1.29)	0.82	1.59 （1.17–2.16）	0.00	1.33 (1.02–1.73)	0.03	1.15 (0.82–1.63)	0.42
Hyperlipidemia	0.92 (0.57–1.48)	0.72	0.92 (0.78–1.09)	0.35	1.02 (0.84–1.25)	0.83	0.79 （0.61–1.03）	0.08	0.89 (0.73–1.08)	0.24	1.05 (0.79–1.37)	0.76
MMSE	1.06 (0.96–1.17)	0.26	1.03 (1.00–1.07)	0.05	1.00 (0.96–1.05)	0.85	1.07 （1.02–1.12）	0.00	1.07 (1.03–1.11)	0.00	1.02 (0.98–1.07)	0.36
GDS-15	1.45 (1.32–1.60)	<.001	1.43 (1.38–1.48)	<.001	1.41 (1.35–1.47)	<.001	1.47 （1.40–1.55）	<.001	1.46 (1.40–1.53)	<.001	1.37 (1.31–1.45)	<.001

Supplementary Table 7. Odds of life satisfaction based on social activity and stratified analysis before imputation.

	Living status				Sex				Age			
	Living alone		Not living alone		Female		Male		Age 75>		Age 75≦	
	OR (95% CI)	p value	OR (95% CI)	p value	OR (95% CI)	p value	OR (96% CI)	p value	OR (95% CI)	p value	OR (96% CI)	p value
**Social activity**												
Low	Reference		Reference		Reference		Reference		Reference		Reference	
Moderate	0.51 (0.30–0.89)	0.02	0.71 (0.59–0.85)	<.001	0.79 (0.63–0.98)	0.04	0.57 (0.44–0.75)	<.001	0.78 (0.63–0.96)	0.02	0.55 (0.40–0.75)	<.001
High	0.51 (0.30–0.87)	0.01	0.68 (0.56–0.81)	<.001	0.69 (0.55–0.88)	0.00	0.61 (0.47–0.79)	<.001	0.71 (0.58–0.96)	0.00	0.61 (0.45–0.84)	0.00
Not living alone					1.06 (0.80–1.40)	0.70	1.19 (0.79–1.79)	0.42	1.00 (0.74–1.34)	0.97	1.13 (0.79–1.62)	0.51
Gender (Male)	1.61 (0.97–2.67)	0.07	1.31 (1.121.52)	<.001					1.26 (1.06–1.50)	0.01	1.51 (1.15–1.96)	0.00
Age	0.95 (0.92–0.99)	0.01	0.94 (0.93–0.95)	<.001	0.93 (0.92–0.95)	<.001	0.95 (0.93–0.97)	<.001				0.88
Year of education	0.99 (0.89–1.10)	0.83	0.97 (0.94–1.01)	0.105	0.99 (0.94–1.04)	0.72	0.96 (0.92–1.00)	0.04	0.99 (0.95–1.03)	0.62	1.01 (0.95–1.07)	
Number of medication	1.07 (0.96–1.18)	0.21	1.04 (1.01–1.08)	0.027	1.05 (1.00–1.10)	0.04	1.04 (0.99–1.09)	0.16	1.02 (0.98–1.07)	0.33	1.04 (0.99–1.09)	0.13
Heart disease	0.81 (0.43–1.52)	0.51	0.84 (0.68–1.04)	0.111	0.88 (0.66–1.17)	0.39	0.79 (0.59–1.06)	0.12	0.77 (0.59–1.00)	0.05	0.87 (0.63–1.19)	0.37
Hypertension	0.68 (0.42–1.11)	0.12	0.92 (0.78–1.09)	0.326	0.91 (0.74–1.12)	0.37	0.90 (0.72–1.14)	0.40	0.83 (0.69–1.00)	0.06	1.00 (0.76–1.30)	0.97
Diabetes	1.19 (0.59–2.39)	0.63	1.29 (1.03–1.61)	0.028	1.01 (0.76–1.36)	0.94	1.65 (1.20–2.26)	0.00	1.44 (1.10–1.87)	0.01	1.13 (0.79–1.61)	0.52
Hyperlipidemia	1.02 (0.62–1.67)	0.94	0.91 (0.76–1.08)	0.265	1.03 (0.84–1.27)	0.76	0.76 (0.58–0.99)	0.04	0.88 (0.72–1.07)	0.19	1.05 (0.79–1.40)	0.72
MMSE	1.04 (0.95–1.15)	0.40	1.03 (1.00–1.06)	0.097	1.00 (0.96–1.04)	0.95	1.06 (1.02–1.11)	0.01	1.07 (1.02–1.11)	0.00	1.02 (0.97–1.06)	0.48
GDS–15	1.43 (1.30–1.58)	<.001	1.42 (1.37–1.48)	<.001	1.40 (1.34–1.47)	<.001	1.46 (1.38–1.54)	<.001	1.46 (1.40–1.52)	<.001	1.35 (1.28–1.42)	<.001

Supplementary Table 8. Odds of life satisfaction based on social activity and stratified analysis after imputation.

	**Living status**				**Sex**				**Age**			
	Living alone		Not living alone		Female		Male		Age 75>		Age 75≦	
	OR (95% CI)		OR (95% CI)		OR (95% CI)	p value	OR (96% CI)	p value	OR (95% CI)	p value	OR (96% CI)	p value
**Social activity**												
Low	Reference		Reference		Reference		Reference		Reference		Reference	
Moderate	0.50 (0.29–0.86)	0.01	0.72 (0.60–0.86)	<.001	0.79 (0.63–0.99)	0.04	0.58 (0.44–0.76)	<.001	0.78 (0.64–0.96)	0.02	0.56 (0.41–0.76)	<.001
High	0.51 (0.30–0.86)	0.01	0.68 (0.57–0.81)	<.001	0.70 (0.55–0.88)	0.00	0.60 (0.47–0.78)	<.001	0.71 (0.58–0.87)	<.001	0.62 (0.45–0.84)	0.00
Not living alone					1.05 (0.80–1.38)	0.74	1.12 (0.75–1.68)	0.58	0.97 (0.73–1.30)	0.84	1.11 (0.78–1.58)	0.56
Gender (Male)	1.50 (0.91–2.48)	0.11	1.31 (1.13–1.52)	<.001					1.25 (1.06–1.49)	0.01	1.50 (1.16–1.95)	0.00
Age	0.95 (0.92–0.98)	0.00	0.94 (0.93–0.95)	<.001	0.93 (0.92–0.95)	<.001	0.95 (0.93–0.96)	<.001				
Year of education	1.00 (0.90–1.11)	1.00	0.97 (0.94–1.00)	0.08	0.99 (0.95–1.04)	0.82	0.95 (0.91–1.00)	0.03	0.99 (0.95–1.03)	0.59	1.00 (0.95–1.06)	0.97
Number of medication	1.06 (0.96–1.17)	0.28	1.04 (1.00–1.08)	0.04	1.05 (1.00–1.09)	0.04	1.03 (0.98–1.09)	0.22	1.02 (0.98–1.07)	0.36	1.03 (0.98–1.09)	0.19
Heart disease	0.85 (0.46–1.59)	0.61	0.88 (0.71–1.09)	0.24	0.93 (0.71–1.23)	0.62	0.83 (0.62–1.11)	0.21	0.80 (0.62–1.04)	0.10	0.92 (0.68–1.25)	0.61
Hypertension	0.75 (0.47–1.21)	0.24	0.94 (0.80–1.10)	0.42	0.93 (0.76–1.14)	0.48	0.94 (0.74–1.18)	0.56	0.84 (0.69–1.01)	0.06	1.06 (0.82–1.38)	0.65
Diabetes	1.10 (0.56–2.18)	0.77	1.26 (1.01–1.57)	0.04	0.99 (0.74–1.32)	0.93	1.60 (1.18–2.18)	0.00	1.35 (1.04–1.76)	0.02	1.17 (0.83–1.66)	0.38
Hyperlipidemia	0.98 (0.60–1.58)	0.92	0.92 (0.78–1.09)	0.34	1.03 (0.84–1.26)	0.79	0.78 (0.60–1.02)	0.07	0.90 (0.74–1.09)	0.28	1.04 (0.79–1.36)	0.81
MMSE	1.04 (0.94–1.15)	0.45	1.03 (1.00–1.06)	0.10	1.00 (0.96–1.04)	0.92	1.06 (1.02–1.11)	0.01	1.06 (1.02–1.10)	0.00	1.02 (0.98–1.07)	0.34
GDS-15	1.43 (1.30–1.58)	<.001	1.41 (1.36–1.47)	<.001	1.40 (1.33–1.46)	<.001	1.45 (1.37–1.52)	<.001	1.45 (1.39–1.52)	<.001	1.34 (1.27–1.41)	<.001
